# The prognostic significance of CXCR4 and SDF-1 in differentiated thyroid cancer depends on CD8+ density

**DOI:** 10.1186/s12902-022-01204-2

**Published:** 2022-11-23

**Authors:** Alexander Wilhelm, Isabelle Lemmenmeier, Alexandros Lalos, Alberto Posabella, Venkatesh Kancherla, Salvatore Piscuoglio, Tarik Delko, Markus von Flüe, Kathrin Glatz, Raoul André Droeser

**Affiliations:** 1 Department of Surgery, Clarunis, St. Clara Hospital and University Hospital Basel, University of Basel, Basel, Postfach, 4002, Switzerland; 2 Department of Surgery, University of California, San Francisco, CA USA; 3 Institute of Pathology, University Hospital Basel, University of Basel, Basel, Switzerland; 4 Visceral Surgery Research Laboratory, Clarunis, Department of Biomedicine, Basel, Switzerland; 5 Department of Surgery, Hirslanden Hospital St. Anna, Lucerne, Switzerland

**Keywords:** Differentiated thyroid cancer, Tumor microenvironment, Chemokines, CXCR4, SDF-1, CD8

## Abstract

**Background:**

Tumor infiltration with cytotoxic CD8+ T-cells is associated with a favorable outcome in several neoplasms, including thyroid cancer. The chemokine axis CXCR4/SDF-1 correlates with more aggressive tumors, but little is known concerning the prognostic relevance in relation to the tumor immune microenvironment of differentiated thyroid cancer (DTC).

**Methods:**

A tissue microarray (TMA) of 37 tumor specimens of primary DTC was analyzed by immunohistochemistry (IHC) for the expression of CD8+, CXCR4, phosphorylated CXCR4 and SDF-1. A survival analysis was performed on a larger collective (*n* = 456) at RNA level using data from The Cancer Genome Atlas (TCGA) papillary thyroid cancer cohort.

**Results:**

Among the 37 patients in the TMA-cohort, the density of CD8+ was higher in patients with less advanced primary tumors (median cells/TMA-punch: 12.5 (IQR: 6.5, 12.5) in T1–2 tumors vs. 5 (IQR: 3, 8) in T3–4 tumors, *p* = 0.05). In the TCGA-cohort, CXCR4 expression was higher in patients with cervical lymph node metastasis compared to N0 or Nx stage (CXCR4^high/low^ 116/78 vs. 97/116 vs. 14/35, respectively, *p* = 0.001). Spearman’s correlation analysis of the TMA-cohort demonstrated that SDF-1 was significantly correlated with CXCR4 (*r* = 0.4, *p* = 0.01) and pCXCR4 (*r* = 0.5, *p* = 0.002). In the TCGA-cohort, density of CD8+ correlated with CXCR4 and SDF-1 expression (*r* = 0.58, *p* < 0.001; *r* = 0.4, *p* < 0.001). The combined marker analysis of the TCGA cohort demonstrated that high expression of both, CXCR4 and SDF-1 was associated with reduced overall survival in the CD8 negative TCGA cohort (*p* = 0.004).

**Conclusion:**

These findings suggest that the prognostic significance of CXCR4 and SDF-1 in differentiated thyroid cancer depends on the density of CD8 positive T-lymphocytes. Further studies with larger sample sizes are needed to support our findings and inform future investigations of new treatment and diagnostic options for a more personalized approach for patients with differentiated thyroid cancer.

## Introduction

Inflammation is an essential component of malignancies influencing the biological behavior of cancer cells. The presence of CD8+ activated T lymphocytes is correlated with a favorable outcome in a variety of solid tumors, including thyroid cancer [1, 2]. The CXC chemokine receptor 4 (CXCR4) is a G protein-coupled chemokine receptor which is responsible for tissue development, cell trafficking, cell proliferation and immune response [3]. CXCR4 is highly expressed throughout embryogenesis while under physiological conditions, is rarely present in somatic adult tissues [4]. Several cancer types show a significant overexpression of CXCR4, including thyroid cancer [4]. Stromal-Derived Factor 1 (SDF-1), also termed C-X-C motif chemokine ligand 12 (CXCL12), acts as a potent chemoattractant for CXCR4-expressing cells [5]. Underlining the importance during the development of distant metastasis, SDF-1 is widely expressed in common metastatic sites like lymph nodes, liver, lung and bone [4]. CXCR4 positive cells follow a chemokine gradient to SDF-1-expressing tissues and activation of CXCR4 via binding of SDF-1 leads to the upregulation of intracellular signalling pathways resulting in proliferation and enhanced survival [6, 7]. CXCR7 is a co-receptor that leads to enhanced SDF-1-mediated signalling [8].

In thyroid cancer, the CXCR4/CXCR7/SDF-1 axis has been investigated gaining importance in the understanding of the progression of thyroid cancer and metastasis development. Werner et al. showed that it functions as key regulator of epithelial–mesenchymal transition (EMT) in follicular thyroid cancer (FTC) and medullary thyroid cancer (MTC) [9, 10]. According to Zhu et al., SDF-1 is particularly expressed in papillary thyroid cancer (PTC), activation of CXCR4 via SDF-1 contributes to thyroid cancer development via regulation of cancer cell migration and invasion [11]. He et al. demonstrated that CXCR4 expression was higher in undifferentiated and medullary thyroid cancer than in differentiated thyroid cancer [12]. In PTC, CXCR4 overexpression was shown to be correlated with a higher rate of lymph node metastasis [13]. Similarly, Torregrossa et al. demonstrated a strong association of CXCR4 with BRAF mutation and neoplastic infiltration, indicating that CXCR4 expression induced by oncogenic activation can be crucial for a more aggressive tumor behaviour [14].

These studies suggest that CXCR4/SDF-1 is correlated with more aggressive thyroid cancers that have a worse prognosis. Therefore, the aim of this study was to explore the clinicopathological role of CXCR4 and SDF-1 with respect to the tumor immune microenvironment in differentiated thyroid carcinoma on a protein and RNA level.

## Methods

### Tissue microarray and immunohistochemistry

A tissue microarray (TMA) was conducted on 37 patients with primary DTC. All methods were performed in compliance with applicable guidelines and regulations. We have described TMA construction in a previous study [15]. The TMAs were generated by specialists of the Pathology Biobank at the University Hospital of Basel (Basel, Switzerland). Formalin-fixed, paraffin-embedded primary DTC tissue blocks were used as donor blocks. Tissue cylinders with a diameter of 1 mm were punched from morphologically representative areas of each donor block and brought into one recipient paraffin block (30 × 25 mm). Each punch was taken from the center of the tumor in an area without necrosis, so that each TMA site contained more than 50% tumor cells.

Standard indirect immunoperoxidase procedures were utilized (IHC; ABC-Elite, Vector Laboratories, Burlingame, CA). Slides were dewaxed and rehydrated in distilled water. Subsequently, endogenous peroxidase activity was blocked using 0.5% H_2_O_2_. Sections were incubated with 10% normal goat serum (DakoCytomation, Carpinteria, CA) for 20 min and incubated with primary antibody at room temperature. Primary antibodies specific for CD8 (Ventana 790–4460, ready to use), pCXCR4 (Abcam ab74012, 1:200 dilution), CXCR4 (Epitomics 31,081, 1:3200 dilution), and SDF-1 (Abcam ab9797, 1:100 dilution) were incubated at room temperature for 16, 32, 16, and 20 minutes, respectively. Afterwards, these were incubated with peroxidase-labeled secondary antibody (DakoCytomation) for 30 min at room temperature. To visualize the antigen, slides were submerged in 3-amino-9-ethylcarbazole plus substrate chromogen (DakoCytomation) for 30 minutes and then counterstained with Gill’s hematoxylin.

Tumour-infiltrating lymphocytes (TIL) were evaluated by immunohistochemistry (CD8). CD8 positive cells within the tumor were considered as TILs. The number of CD8 positive tumor-infiltrating T-lymphocytes was counted in one representative high-power field of each TMA spot.

Clinico-pathological data were collected retrospectively, including patient age, sex, tumor diameter in mm, histologic subtype, pT-stage, pN-stage, and pM-stage (Table 1).

**Table 1:** Characteristics of the TMA (*n* = 37) and TCGA cohort (*n* = 456)

Characteristic	WDTC (TMA-cohort) (*n* = 37)	PTC (TCGA-cohort) (*n* = 456)
**Age** (years, mean, range)	45.4 (15–81)	46.8 (15–88)
**Age**		
< 55	27 (73%)	309 (68%)
≥ 55	10 (27%)	147 (32%)
**Sex (N, %)**		
Male	14 (38%)	121 (27%)
Female	23 (62%)	335 (73%)
**Tumor size** in mm, mean (range)	30.8 (8–61)	29.2 (1–94)
**Histologic subtype (N, %)**		
PTC, classic	12 (32%)	355 (78%)
PTC, follicular variant	12 (32%)	101 (22%)
PTC, other variants	3 (8%)	
FTC	6 (16%)	
Oncocytic carcinoma	4 (11%)	
**pT-Classification (N, %)**		
T1–2	25 (68%)	294 (65%)
T3–4	12 (32%)	160 (35%)
**pN-Classification (N, %)**		
N0	12 (32%)	213 (47%)
N1	7 (19%)	194 (43%)
Nx	18 (49%)	49 (10%)
**pM-Classification (N, %)**		
M0	0 (0%)	254 (56%)
M1	0 (0%)	9 (2%)
Mx	37 (100%)	192 (42%)

### Analysis of TCGA data

CD8A, SDF-1 and CXCR4 fragments per kilo base of transcript per million mapped fragments (FPKM) gene-level expression data from The Cancer Genome Atlas (TCGA) papillary thyroid cancer (*n* = 456) was obtained from human protein atlas [16]. Tumor samples were classified into high and low groups based on the median threshold. Utilizing these data, single marker analyses and combined marker survival analysis was performed. Additionally, a spearman’s correlation was calculated between the genes. Clinicopathologic features information was obtained from The Cancer Genome Atlas (Table 1) [17].

### Statistical analysis

In the TMA cohort, the median histoscores were calculated by multiplying the percentage of positive cells with the semiquantitative staining intensity score (0–3) for pCXCR4, CXCR4, and SDF-1. The median histoscore for each biomarker and the number of tumor-infiltrating lymphocytes determined the cut-off values used to classify tumor specimens with low or high protein expression and immune cell infiltration. Threshold values for pCXCR4, CXCR4, and SDF-1 histoscore was 30, 50, and 180, respectively. Threshold value for high immune cell infiltration was 8 CD8+ cells/TMA-punch. Statistical comparisons between categorical variables were performed using the Kruskal-Wallis and Wilcoxon rank sum tests for the TMA cohort and the χ2 test for the TCGA cohort. Kaplan–Meier method and log-rank test were used to perform survival analysis. Stratification of expression of the genes for overall survival analysis was based on the median. Further analyses included possible combinations (SDF-1^high^/CXCR4^high^, SDF-1^high^/CXCR4^low^, SDF-1^high^/ CXCR4^low^) and the resulting Kaplan–Meier curves compared with adjusted *p*-value for multiple comparisons according to Benjamini and Hochberg. Association between overall survival and clinical variables was performed using Univariate Cox proportional hazard regression analyses. Variables significant in the univariate Cox regression model were included in multivariate Cox regression analysis. The prognostic impact on survival was assessed using hazard ratios (HR) and 95% confidence intervals (CI). All tests were two-sided, and *p* < 0.05 was considered statistically significant. Statistical analyses were made using STATA software version 13 (StataCorp, College Station, TX, USA) and with the Statistical Package Software R (version 4.0.2, (http://r-project.org).

## Results

### Patient and tumor characteristics – TMA Cohort

We included 37 patients with differentiated thyroid cancer in our TMA-cohort (Table 1). Mean age was 45.4 years (range 15–81). Of the total 37 patients, 62% were female. Mean tumor size was 30.8 mm (range 8–61). In 32% of the patients, histologic diagnosis was classic PTC, 32% had follicular variant of PTC, followed by 16% with FTC, 11% with oncocytic thyroid cancer, and 8% with other variants of PTC. Most of the tumors were at pT1/2 stage (68%) and 19% had cervical lymph node metastasis.

### Patient and tumor characteristics – TCGA Cohort

The TCGA dataset provides comprehensive information on 456 PTC specimen (Table 1). Mean age was 46.8 (range 15–88), 73% of patients were female. Mean tumor size was 29.2 mm (range 1–94), most common histologic subtype was classic variant of PTC (78%), followed by 22% with follicular variant of PTC. The majority of the patients were at pT1/2 stage (64%). In 43% of patients, cervival lymph node metastasis was diagnosed, only 2% had distant metastases.

### Association of clinicopathological features with CD8, CXCR4, pCXCR4 and SDF-1 expression

**Fig. 1:**
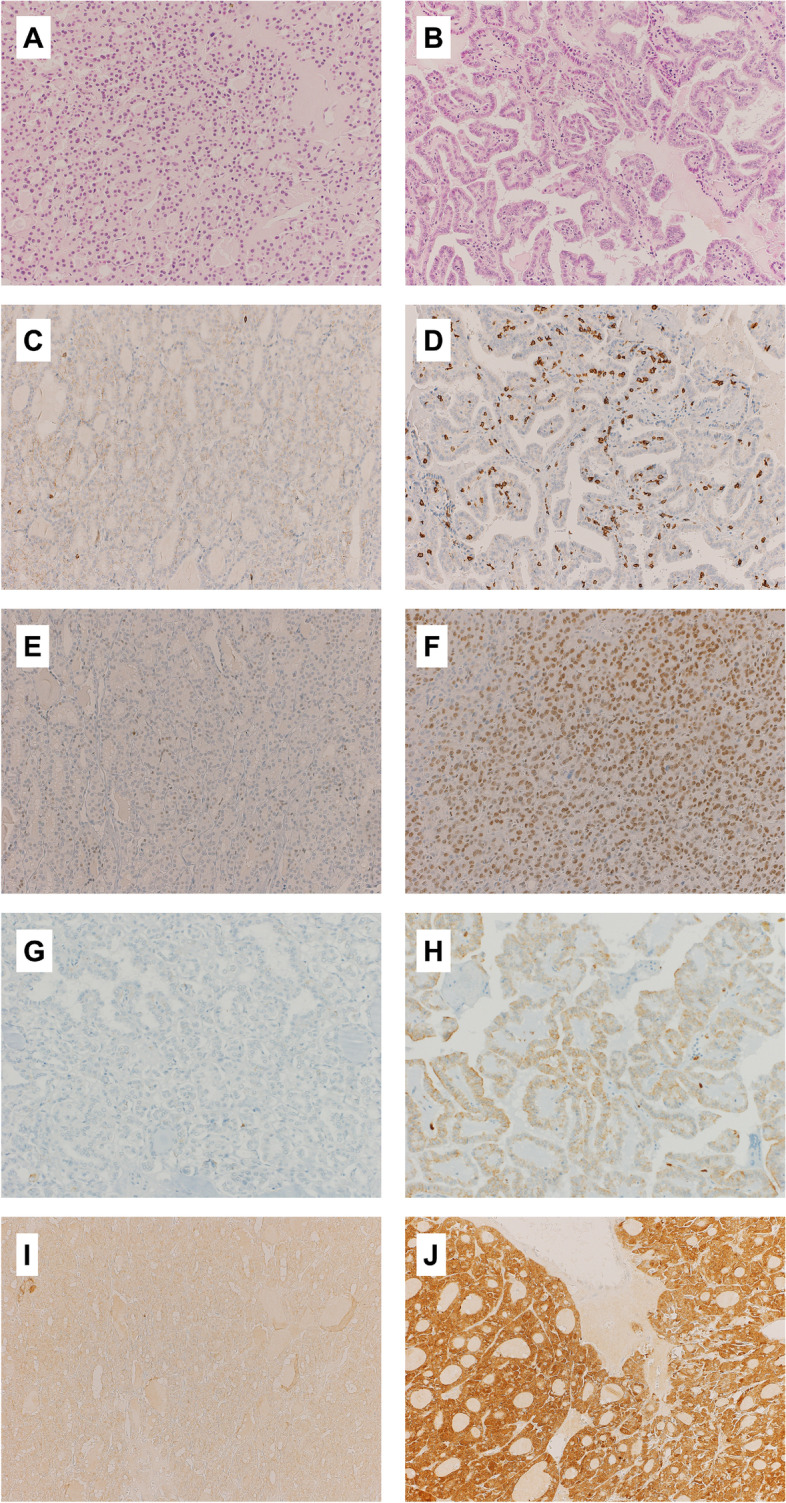
Examples of H&E stainings and immunohistochemical stainings (× 200 magnification) of the tissue microarray: low amount of TILs in a follicular thyroid carcinoma (**A**), high amount of TILs in a papillary thyroid carcinoma, classic type (**B**), with low density of CD8+ cells in a papillary thyroid carcinoma, classic type (**C**), and with high density of CD8+ cells in a follicular thyroid carcinoma (**D**), low expression of CXCR4 (**E**) and high expression of CXCR4 in follicular thyroid carcinomas (**F**), low density of pCXCR4 (**G**) and high density of pCXCR4 (**H**) in papillary thyroid carcinomas of follicular and classic type, respectively, low expression of SDF-1 (**I**) in a follicular thyroid carcinoma and high expression of SDF-1 (**J**) in an oncocytic thyroid carcinoma

In Fig. 1, we illustrate representative histopathological and immunohistochemical pictures of low and high expression of CXCR4, pCXCR4, SDF-1, and with high and low CD8 positive T-cell infiltration.

Table 2 shows the distribution of CD8+, CXCR4, pCXCR4, SDF-1 in relation to the clinicopathological features from the TMA cohort. The density of CD8-positive T lymphocytes was higher in patients with less advanced primary tumors (median cells/TMA-punch: 12.5 (IQR: 6.5, 12.5) in T1–2 tumors vs. 5 (IQR: 3, 8) in T3–4 tumors, *p* = 0.05). There was a non-significant trend toward higher expression of SDF-1 in patients older than 55 years compared to patients younger than 55 years (median histoscore: 200 (IQR: 185, 250) in patients ≥55 years vs. 100 (IQR: 25, 200) in patients < 55 years, *p* = 0.08). The variations of the other biomarkers in relation to the clinicopathological characteristics did not reach statistical significance.

**Table 2:** Association of CD8, CXCR4, pCXCR4, SDF-1 expression with clinicopathological features in DTC (TMA-cohort, *n* = 37)

Characteristic	CD8 cells/TMA punch median (IQR) (*n* = 37)	*p*-value*	CXCR4 histoscore median (IQR) (*n* = 37)	*p*-value*	pCXCR4 histoscore median (IQR) (*n* = 37)	*p*-value*	SDF-1 histoscore median (IQR) (*n* = 37)	*p*-value
**Age at diagnosis**								
< 55	10 (3, 14.5)	0.97	15 (0, 120)	0.21	5 (0, 50)	0.18	100 (25, 200)	0.08
≥ 55	7 (5, 19)		95 (25, 140)		20 (5, 180)		200 (185, 250)	
**Sex (N)**								
Male	6 (3, 12)	0.28	10 (0, 50)	0.16	5 (0, 30)	0.56	180 (100, 200)	0.68
Female	10 (5, 15)		90 (0, 90)		10 (0,70)		100 (30, 300)	
**Histologic subtype (N)**								
PTC, classic	13.5 (4.5, 22.5)	0.61	20 (0, 140)	0.61	20 (0, 95)	0.87	100 (15, 185)	0.06
PTC, follicular variant	9 (4, 13)		50 (12.5, 130)		5 (0, 50)		140, 10, 200)	
PTC, other variants	8 (5, 15)		0 (0, 140)		10 (5, 180)		200 (100, 300)	
FTC	6 (5, 10)		90 (10, 270)		10 (0, 30)		180 (100, 200)	
Oncocytic carcinoma	6 (1.5, 13)		17.5 (2.5, 60)		50 (0, 140)		300 (250, 300)	
**pT-Classification (N)**								
T1–2	12.5 (6.5, 15.5)	0.05	40 (0, 150)	0.65	5 (0, 50)	0.42	180 (90, 250)	0.71
T3–4	5 (3, 8)		20 (0, 100)		10 (0, 140)		145 (15, 200)	
**pN-Classification (N)**								
N0	11 (7, 15)	0.55	70 (2.5, 130)	0.33	5 (0, 70)	0.33	180 (15, 250)	0.62
N1	5 (3, 20)		0 (0, 160)		0 (0, 10)		100 (90, 180)	
Nx	8 (3, 13)		30 (10, 140)		10 (0, 140)		190 (100, 200)	

*Kruskal-Wallis or Wilcoxon rank sum test as appropriate

Table 3 demonstrates the association of CD8+, CXCR4, and SDF-1 in relation to the clinicopathological variables in the PTC-cohort of the TCGA-database. Patient age was significantly associated with differences in the expression of CD8+ and CXCR4. CD8+ density was higher in patients younger than 55 years compared to older patients (CD8^high/low^ 160/149 in patients < 55 years vs. 58/89 in patients ≥55 years, *p* = 0.014). Younger patients had higher expression of CXCR4 compared to patients ≥55 years (CXCR4^high/low^ 170/139 in patients < 55 years vs. 57/90 in patients ≥55 years, *p* = 0.001). In contrast, there was a non-significant trend of a higher SDF-1 expression in patients ≥55 years compared to patients < 55 years (SDF-1^high/low^ 82/65 in patients ≥55 years vs. 144/165 in patients < 55 years, *p* = 0.067). In patients with cervical lymph node metastasis, CXCR4 expression was higher compared to those with N0 or Nx stage (CXCR4^high/low^ 116/78 vs. 97/116 vs. 14/35, respectively, *p* = 0.001). The expression of CXCR4 and SDF-1 was higher in classic PTC compared to follicular variant of PTC (CXCR4^high/low^ 188/167 vs. 39/62, *p* = 0.011; SDF-1^high/low^ 190/165 vs. 36/65, *p* = 0.002, respectively).

**Table 3:** Association of CD8, CXCR4, SDF-1 expression with clinicopathological features in PTC (TCGA-cohort, *n* = 456)

Characteristic	CD8 high/low (*n* = 218/238)	*p*-value*	CXCR4 high/low (*n* = 227/229)	*p*-value*	SDF-1 high/low (*n* = 226/230)	*p*-value*
**Age at diagnosis**						
< 55	160/149	**0.014**	170/139	**0.001**	144/165	0.067
≥ 55	58/89		57/90		82/65	
**Sex (N, %)**						
Male	51/70	0.146	58/63	0.635	62/59	0.667
Female	167/168		169/166		164/171	
**Histologic subtype**						
PTC, classic	177/178	0.101	188/167	**0.011**	190/165	**0.002**
PTC, follicular variant	41/60		39/62		36/65	
**pT-Classification**						
T1–2	138/156	0.620	142/152		0.392	**0.032**
T3–4	79/81		84/76		68/92	
**pN-Classification**						
N0	101/112	**0.009**	97/116	**0.001**	115/98	0.204
N1	103/91		116/78		88/106	
Nx	14/35		14/35		23/26	
**UICC stage**						
I/II	153/163	0.157	160/156	0.655	157/159	0.25
III	47/43		46/44		49/41	
IV	17/31		21/27		19/29	

*χ2 test

### Spearman’s correlation analysis of CXCR4, pCXCR4, SDF-1, and CD8+ T-cell density

A spearman’s correlation analysis evaluated the relation of SDF-1 protein expression with CXCR4, pCXCR4 and CD8+ density in the TMA-cohort (Table 4). Numbers greater than 0.35 indicate a strong correlation. The expressions of CXCR4, pCXCR4 and SDF-1 were strongly interrelated (*r* = 0.43, *p* = 0.010, and *r* = 0.50, *p* = 0.002, respectively). Density of CD8+ tumor-infiltrating lymphocytes showed no correlation with the other proteins.

**Table 4:** Spearman’s correlation analysis of SDF-1 protein expression with CD8 density, and CXCR4, pCXCR4 expression in the TMA cohort

	SDF-1-expression	CD8-expression	CXCR4-expression	pCXCR4-expression
**SDF-1-expression**	1.000	0.06 (*p* = 0.74)	0.43 (*p* = 0.010)	0.50 (*p* = 0.002)
**CD8-expression**		1.000	0.26 (*p* = 0.125)	0.23 (*p* = 0.181)
**CXCR4-expression**		–	1.000	0.55 (*p* < 0.001)
**pCXCR4-expression**		–	–	1.000

In the TCGA cohort, CD8+ density correlated strongly with SDF-1 and CXCR4 expression (*r* = 0.40, *p* < 0.001, and *r* = 0.58, *p* < 0.001, respectively) (Table 5). Furthermore, the correlation of CXCR4 and SDF-1 expression was moderate (*r* = 0.33, *p* < 0.001).

**Table 5:** Spearman’s correlation analysis of SDF-1 protein expression with CXCR4 and CD8 in the TCGA cohort

	SDF-1-expression	CD8-expression	CXCR4-expression
**SDF-1-expression**	1.000	0.40 (*p* < 0.001)	0.33 (*p* < 0.001)
**CD8-expression**	–	1.000	0.58 (*p* < 0.001)
**CXCR4-expression**	–	–	1.000

### Survival analysis

Cox proportional hazard regression analysis (Table 6) demonstrated that more advanced T-stage (pT3/4 vs. pT1/2: HR = 3.3; 95%CI 1.1–9.6, *p* = 0.03) and M-stage (pM1 vs pM0/x: HR = 4.7, 95%CI 1.1–21, *p* = 0.04) were significantly associated with a higher risk of death in the univariate analysis. In the multivariate analysis, only T-stage (HR = 3.1, 95%CI 1.1–8.9, *p* = 0.04) was significantly associated with a worse prognosis. The combination of CXCR4 and SDF-1 expression did not significantly influence the oncological outcome.

**Table 6:** Uni- and multivariable Hazard Cox regression survival analysis in the TCGA-cohort (*n* = 456)

Characteristic	Univariable Analysis			Multivariable Analysis		
	Hazard Ratio	95% CI	***P*** value	Hazard Ratio	95% CI	***P*** value
**Age** (≥55 vs < 55)	3.1		1	–		
**Sex** (Male vs female)	2.1	(0.75–5.7)	0.16	–		
**pT** (high vs. low)	3.3	(1.1–9.6)	**0.03**	3.1	(1.1–8.9)	**0.04**
**pN** (1 vs. 0/x)	1.2	(0.45–3.2)	0.73	–		
**pM** (1 vs. 0/x)	4.7	(1.1–21)	**0.04**	3.7	(0.8–16)	0.08
SDF1^low^/CXCR4^low^ vs. **SDF1**^**high**^**/CXCR4**^**high**^	0.7	(0.22–2.2)	0.54	–		
SDF1^low^/CXCR4^low^ vs. **SDF1**^**low**^**/CXCR4**^**high**^	1.9	(0.64–1.1)	0.24	–		
SDF1^low^/CXCR4^low^ vs. **SDF1**^**high**^**/CXCR4**^**low**^	3.6	(0.97–1)	0.86	–		

Variables significant (*p* < 0.05) in univariate analyses were included in multivariate Cox regression analysis

Analyzing only the CD8-negative cohort (Table 7), the combined high expression of CXCR4 and SDF-1 was a significant prognostic factor (SDF-1^low^/CXCR4^low^ vs. SDF-1^high^/CXCR4^high^: HR = 0.21, 95%CI 0.05–0.84, *p* = 0.03) in the univariate analysis. T-stage (HR = 7.3, 95%CI 1.5–34, *p* = 0.01), and M-stage (HR = 11, 95%CI 2.3–52, *p* = 0.002) were also independently associated with a poor prognosis. In the multivariate analysis, higher T-stage (HR = 9.7, 95%CI 1.1–84, *p* = 0.03) and high expression of CXCR4 and SDF-1 (SDF-1^low^/CXCR4^low^ vs. SDF-1^high^/CXCR4^high^: HR = 0.20, 95%CI 0.04–0.70, *p* = 0.02) were associated with a higher risk of death.

**Table 7:** Uni- and multivariable Hazard Cox regression survival analysis in the CD8 negative TCGA-cohort (*n* = 238)

Characteristic	Univariable Analysis			Multivariable Analysis		
	Hazard Ratio	95% CI	***P*** value	Hazard Ratio	95% CI	***P*** value
**Age** (≥55 vs < 55)	–		1	**–**		
**Sex** (Male vs female)	1.1	(0.29–4.4)	0.86	–		
**pT** (high vs. low)	7.3	(1.5–34)	**0.01**	9.7	(1.1–84)	**0.03**
**pN** (1 vs. 0/x)	0.94	(0.26–3.4)	0.93	–		
**pM** (1 vs. 0/x)	11	(2.3–52)	**0.002**	4.9	(0.9–26)	0.07
SDF1^low^/CXCR4^low^ vs **SDF1**^**high**^**/CXCR4**^**high**^	0.21	(0.05–0.84)	**0.03**	0.2	(0.04–0.7)	**0.02**
SDF1^low^/CXCR4^low^ vs **SDF1**^**low**^**/CXCR4**^**high**^	1.4	(0.25–7.5)	0.71	–		
SDF1^low^/CXCR4^low^ vs. **SDF1**^**high**^**/CXCR4**^**low**^	–		1	–		

Variables significant (*p* < 0.05) in univariate analyses were included in multivariate Cox regression analysis

**Fig. 2:**
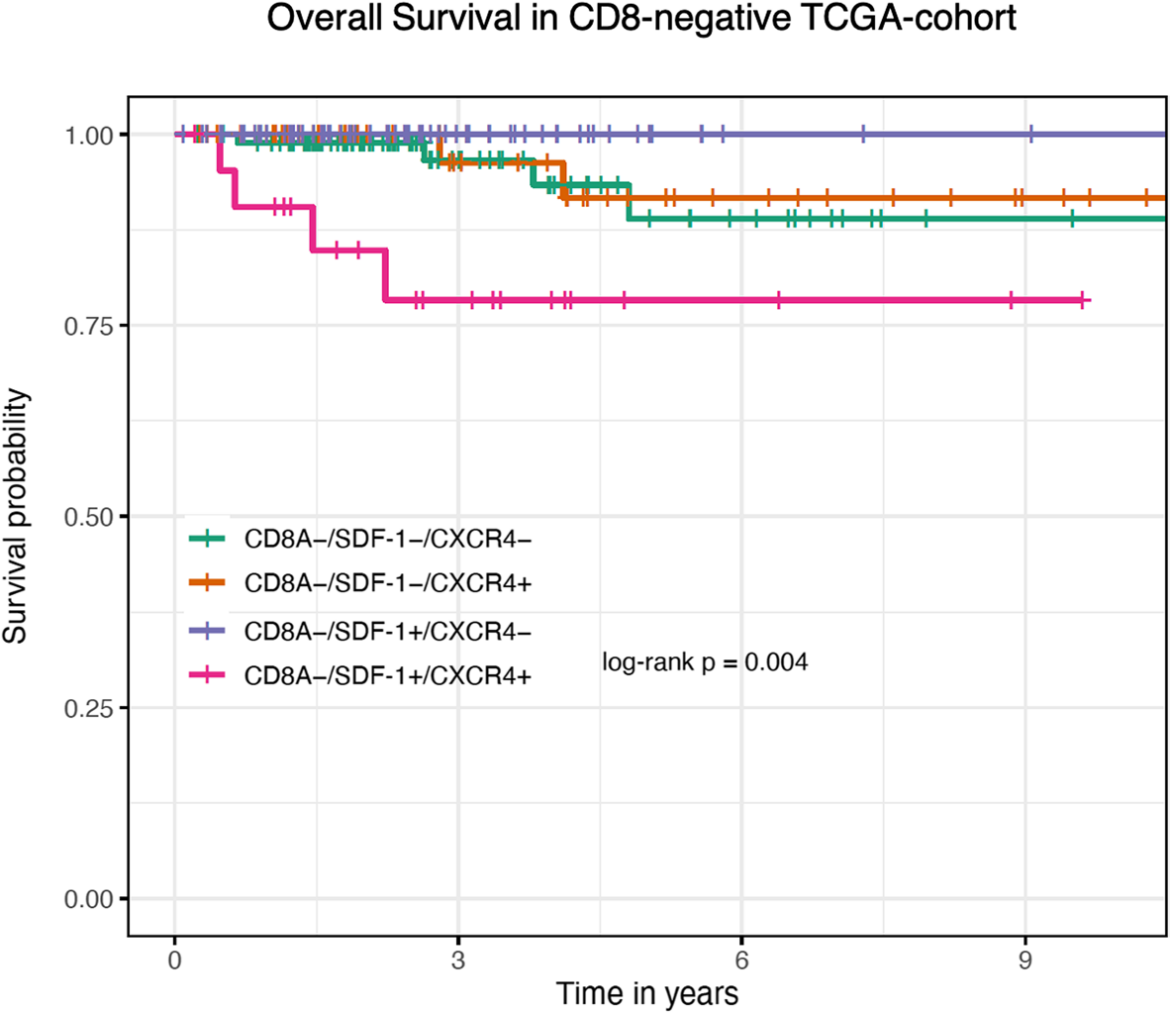
Kaplan-Meier Overall survival curve in the CD8-negative cohort of the TCGA-cohort (*n* = 238)

Furthermore, these findings are illustrated in the Kaplan-Meier survival analysis. In the CD8-negative cohort, patients with high expression of both CXCR4 and SDF-1 have a significantly decreased overall survival compared to other expression combinations of the two biomarkers (log-rank *p*-value = 0.004; Fig. 2).

## Discussion

To our knowledge, this is the first study investigating the prognostic significance of CXCR4 and SDF-1 in differentiated thyroid cancer in relation to the density of CD8 positive T-lymphocytes. The expression of CD8 positive T-cells was higher in patients < 55 years old and in less advanced T-stages. CXCR4 expression was higher in patients with classic PTC compared to follicular variant and in patients with cervical lymph node metastasis. SDF-1 density was higher in classic PTC. In the CD8-negative cohort, the combined high expression of CXCR4 and its ligand SDF-1 was associated with decreased overall survival, suggesting that the detrimental effect of CXCR4/SDF-1 depends on the density of tumor infiltrating CD8-positive T lymphocytes.

The tumor immune microenvironment regulates responses of immune cells and play a critical role in the suppression of cancer development and cancer spread [18]. Due to their ability to destroy cancer cells, cytotoxic CD8 positive T-lymphocytes are among the most important inhibitors of carcinogenesis [19]. Blessin NC et al. provides a wide-ranging summary of the differences of CD8-positivity in a variety of normal tissues and cancer types [20]. According to their analysis, there are two types of malignant neoplasms that have a high density of tumor-infiltrating CD8 positive T-lymphocytes: on the one hand, aggressive tumors such as anaplastic thyroid cancer, and on the other hand, tumors that are known to respond to immunotherapy, such as malignant melanoma or clear cell renal cancer [20]. This supports the theory that a high density of CD8+ T-cells increases the likelihood of response to such therapy [21]. Additionally, recent studies demonstrated decreased CD8+ density in more advanced tumor stages in breast cancer and papillary thyroid cancer [22, 23]. In this study, expression of CD8+ T-lymphocytes was lower in older patients and in patients with more advanced T-stages, supporting the results of previous studies [22, 23]. Known to be associated with a poor prognosis in papillary thyroid cancer, Yang et al. demonstrated that the density of CD8+ tumor-infiltrating T-lymphocytes was reduced in thyroid cancers with BRAF^V600E^ mutation compared with BRAF^V600E^ wild-type cancers [23].

The majority of drivers of thyroid cancer are known since the first comprehensive analysis of The Cancer Genome Atlas [24]. A variety of immunocompetent cells, cytokines and chemokines are currently under investigation to pave the way towards a more personalized cancer therapy [25, 26]. Similar to other cancers, it is critical to understand the process of thyroid cancer progression in order to improve diagnostic methods and treatment options. CXCR4 is overexpressed and plays a key role in the carcinogenesis in a variety of solid tumors [7]. Activation via binding of SDF-1 initiates different signaling pathways resulting in proliferation, chemotaxis, and site-specific metastasis of CXCR4-expressing cancer cells [27, 28]. Accordingly, the results of the TCGA cohort demonstrated higher expression of CXCR4 and SDF-1 in classic papillary thyroid cancer, reflecting the relative importance of the CXCR4/SDF-1 interaction predominantly in this thyroid cancer histology compared with other thyroid cancer types. According to the literature, high expressions of CXCR4 and SDF-1 were also detected in FTC, but mainly in the sites of distant metastases compared to primary tumors, highlighting its prognostic significance in the process of disseminating cancer cells [9]. Classic PTC metastasize more frequently to regional lymph nodes, so analogous to the literature, our study demonstrated an increased density of CXCR4 in patients with cervical lymph node metastases [29].

The spearman’s analysis of the TMA cohort showed that the expression of SDF-1 and CXCR4, was strongly correlated. The significant correlation of pCXCR4 with both CXCR4 and SDF-1 suggests that binding of SDF-1 leads to substantial receptor phosphorylation of CXCR4 and hence activation of intracellular signaling pathways. In the TCGA cohort, density of CD8+ tumor-infiltrating T-lymphocytes showed a strong correlation with CXCR4 expression, underlining that CXCR4 acts as a potent driver of T-cell migration, similar to a recent study [30].

It has been shown for a variety of cancer types, including papillary thyroid cancer, that high CD8+ density is associated with a favorable outcome [31–37]. CXCR4 and SDF-1 are indicators of more aggressive papillary thyroid cancer types and act as key regulators of local and distant metastasis in various types of thyroid cancer, leading to a poorer prognosis in CXCR4/SDF-1-positive cancers [10, 13, 25]. By combining the evaluation of the chemokine axis CXCR4/SDF-1 in relation to CD8+ density, the survival analysis in this study suggests that the detrimental effect of high CXCR4/SDF-1 expression depends on the density of tumor-infiltrating CD8+ cytotoxic T-lymphocytes. The cox proportional hazard regression analysis of the entire TCGA cohort indicates that the positive predictive value of CD8+ T-cells potentially outweighed the disadvantage of the CXCR4/SDF-1 axis. While neither CXCR4, SDF-1 nor CD8+ density showed a significant prognostic effect alone, in the CD8 negative cohort, the combination of high expression of CXCR4 and SDF-1 was significantly associated with a negative outcome in the uni- and multivariable analysis. The Kaplan-Meier survival analysis displays that the negative predictive significance of high expression of CXR4/SDF-1 may depend on CD8+ density.

These new insights can be valuable in multiple ways and potentially pave the way for new diagnostic and therapeutic opportunities. Based on these findings, further studies are needed to evaluate the prognostic significance of CXCR4/SDF-1 in relation to CD8+ density in different cancers with known prognostic influence of the CXCR4/SDF-1 axis alone, including different types of thyroid cancer. The assumption that the density of lymphocytes in the tumor microenvironment is related to the sensitivity to immune checkpoint inhibitors is well established [38]. Thus, it will be crucial in the treatment of those cancers where analyses of the lymphocyte density indicate that immune therapy might be less effective to develop different therapeutic targets.

We would like to acknowledge the limitations of this study. First, the TMA technology might not be able to detect tumor tissue heterogeneity, especially with the small sample size in our study. However, our group has gained extensive expertise with previous TMA studies, assuring that punches are made out of the tumor center to include at least 50% of tumor cells [39–41]. Second, due to the fact that differentiated thyroid cancer harbors an excellent prognosis compared to other cancers and the small number of cancer specimen in our TMA cohort, we were not able to evaluate the prognostic relevance of CXCR4, pCXCR4, SDF-1 and CD8+ in this cohort. Nevertheless, spearman’s correlation analysis demonstrated that both SDF-1 and CXCR4 expression strongly correlated with pCXCR4 expression, indicating that interaction leads to significant receptor phosphorylation.

## Conclusion

In conclusion, the presented study suggests that the prognostic significance of the combined high expression of CXCR4 and SDF-1 in differentiated thyroid cancer depends on the density of tumor-infiltrating CD8 positive T-lymphocytes. These novel insights may trigger further research to corroborate our findings and can lead to the investigation of new diagnostic and treatment options towards a more personalized approach for differentiated thyroid cancer patients.

## Data Availability

The datasets used and/or analysed during the current study are available from the corresponding author on reasonable request.

## References

[1] Galon J, Mlecnik B, Bindea G, Angell HK, Berger A, Lagorce C (2014). Towards the introduction of the ‘Immunoscore’ in the classification of malignant tumours. J Pathol.

[2] Aghajani MJ, Yang T, Schmitz U, James A, McCafferty CE, de Souza P (2020). Epithelial-to-mesenchymal transition and its association with PD-L1 and CD8 in thyroid cancer. Endocr Connect.

[3] Busillo JMB, J.L. (2007). Regulation of CXCR4 Signaling. Biochim Biophys Acta.

[4] Balkwill F (2004). Cancer and the chemokine network. Nat Rev Cancer.

[5] Wright LM, Maloney W, Yu X, Kindle L, Collin-Osdoby P, Osdoby P (2005). Stromal cell-derived factor-1 binding to its chemokine receptor CXCR4 on precursor cells promotes the chemotactic recruitment, development and survival of human osteoclasts. Bone.

[6] Darash-Yahana M, Pikarsky E, Abramovitch R, Zeira E, Pal B, Karplus R (2004). Role of high expression levels of CXCR4 in tumor growth, vascularization, and metastasis. FASEB J.

[7] Sun X, Cheng G, Hao M, Zheng J, Zhou X, Zhang J (2010). CXCL12 / CXCR4 / CXCR7 chemokine axis and cancer progression. Cancer Metastasis Rev.

[8] Levoye A, Balabanian K, Baleux F, Bachelerie F, Lagane B (2009). CXCR7 heterodimerizes with CXCR4 and regulates CXCL12-mediated G protein signaling. Blood.

[9] Werner TA, Forster CM, Dizdar L, Verde PE, Raba K, Schott M (2018). CXCR4/CXCR7/CXCL12-Axis in Follicular Thyroid Carcinoma. J Cancer.

[10] Werner TA, Forster CM, Dizdar L, Verde PE, Raba K, Schott M (2017). CXCR4/CXCR7/CXCL12 axis promotes an invasive phenotype in medullary thyroid carcinoma. Br J Cancer.

[11] Zhu X, Bai Q, Lu Y, Lu Y, Zhu L, Zhou X (2016). Expression and function of CXCL12/CXCR4/CXCR7 in thyroid cancer. Int J Oncol.

[12] He X, Wei Q, Zhang X, Xiao J, Jin X, Zhu Y (2010). Immunohistochemical expression of CXCR4 in thyroid carcinomas and thyroid benign lesions. Pathol Res Pract.

[13] Wang N, Luo HJ, Yin GB, Dong CR, Xu M, Chen GG (2013). Overexpression of HIF-2α, TWIST, and CXCR4 is associated with lymph node metastasis in papillary thyroid carcinoma. Clin Dev Immunol.

[14] Torregrossa L, Giannini R, Borrelli N, Sensi E, Melillo RM, Leocata P (2012). CXCR4 expression correlates with the degree of tumor infiltration and BRAF status in papillary thyroid carcinomas. Mod Pathol.

[15] Sauter G, Simon R, Hillan K (2003). Tissue microarrays in drug discovery. Nat Rev Drug Discov.

[16] Uhlen M, Zhang C, Lee S, Sjöstedt E, Fagerberg L, Bidkhori G, et al. A pathology atlas of the human cancer transcriptome. Science. 2017;357(6352). 10.1126/science.aan2507. 10.1126/science.aan2507 28818916

[17] Grossman RL, Heath AP, Ferretti V, Varmus HE, Lowy DR, Kibbe WA (2016). Toward a Shared Vision for Cancer Genomic Data. N Engl J Med.

[18] Weber F (2014). Lymphocytes and thyroid cancer: more to it than meets the eye?. Endocr Relat Cancer.

[19] Grivennikov SI, Karin M (2010). Inflammation and oncogenesis: a vicious connection. Curr Opin Genet Dev.

[20] Blessin NC, Spriestersbach P, Li W, Mandelkow T, Dum D, Simon R (2020). Prevalence of CD8(+) cytotoxic lymphocytes in human neoplasms. Cell Oncol (Dordr).

[21] French JD (2020). Immunotherapy for advanced thyroid cancers - rationale, current advances and future strategies. Nat Rev Endocrinol.

[22] Meng S, Li L, Zhou M, Jiang W, Niu H, Yang K (2018). Distribution and prognostic value of tumorinfiltrating T cells in breast cancer. Mol Med Rep.

[23] Yang Z, Wei X, Pan Y, Xu J, Si Y, Min Z (2021). A new risk factor indicator for papillary thyroid cancer based on immune infiltration. Cell Death Dis.

[24] Cancer Genome Atlas Research N (2014). Integrated genomic characterization of papillary thyroid carcinoma. Cell.

[25] Coperchini F, Croce L, Marino M, Chiovato L, Rotondi M (2019). Role of chemokine receptors in thyroid cancer and immunotherapy. Endocr Relat Cancer.

[26] Fallahi P, Ferrari SM, Piaggi S, Luconi M, Cantini G, Gelmini S (2018). The paramount role of cytokines and chemokines in papillary thyroid cancer: a review and experimental results. Immunol Res.

[27] Lippitz BE (2013). Cytokine patterns in patients with cancer: a systematic review. Lancet Oncol.

[28] Teicher BA, Fricker SP (2010). CXCL12 (SDF-1)/CXCR4 pathway in cancer. Clin Cancer Res.

[29] Cao X, Zhu J, Li X, Ma Y, He Q. Expression of CXCR4 and CXCR7 in papillary thyroid carcinoma and adjacent tissues and their relationship with pathologic indicators of tumor aggressiveness. Endocr J. 2021. 10.1507/endocrj.EJ21-0076. 10.1507/endocrj.EJ21-0076 34588386

[30] Goedhart M, Gessel S, van der Voort R, Slot E, Lucas B, Gielen E (2019). CXCR4, but not CXCR3, drives CD8(+) T-cell entry into and migration through the murine bone marrow. Eur J Immunol.

[31] Peng GL, Li L, Guo YW, Yu P, Yin XJ, Wang S, et al. CD8+ cytotoxic and FoxP3+ regulatory T lymphocytes serve as prognostic factors in breast cancer. Am J Transl Res. 2019;11(8):5039–53. PMC6731430 31497220

[32] van der Leun AM, Thommen DS, Schumacher TN (2020). CD8(+) T cell states in human cancer: insights from single-cell analysis. Nat Rev Cancer.

[33] Xu X, Tan Y, Qian Y, Xue W, Wang Y, Du J (2019). Clinicopathologic and prognostic significance of tumor-infiltrating CD8+ T cells in patients with hepatocellular carcinoma: A meta-analysis. Medicine (Baltimore).

[34] Goode EL, Block MS, Kalli KR, Vierkant RA, Chen W, Fogarty ZC (2017). Dose-Response Association of CD8+ Tumor-Infiltrating Lymphocytes and Survival Time in High-Grade Serous Ovarian Cancer. JAMA Oncol.

[35] Kim SH, Go SI, Song DH, Park SW, Kim HR, Jang I (2019). Prognostic impact of CD8 and programmed death-ligand 1 expression in patients with resectable non-small cell lung cancer. Br J Cancer.

[36] Rabold K, Gielen PR, Kers-Rebel ED, Netea MG, Smit JWA, Adema GJ (2019). T-Cell lymphopenia in patients with advanced thyroid carcinoma is associated with poor prognosis. Oncologist.

[37] Aghajani MJ, Yang T, McCafferty CE, Graham S, Wu X, Niles N (2018). Predictive relevance of programmed cell death protein 1 and tumor-infiltrating lymphocyte expression in papillary thyroid cancer. Surgery.

[38] Chen DS, Mellman I (2017). Elements of cancer immunity and the cancer-immune set point. Nature.

[39] Däster S, Eppenberger-Castori S, Hirt C, Soysal SD, Delko T, Nebiker CA (2015). Absence of myeloperoxidase and CD8 positive cells in colorectal cancer infiltrates identifies patients with severe prognosis. Oncoimmunology.

[40] Lalos A, Tulek A, Tosti N, Mechera R, Wilhelm A, Soysal S (2021). Prognostic significance of CD8+ T-cells density in stage III colorectal cancer depends on SDF-1 expression. Sci Rep.

[41] Weixler B, Renetseder F, Facile I, Tosti N, Cremonesi E, Tampakis A (2017). Phosphorylated CXCR4 expression has a positive prognostic impact in colorectal cancer. Cell Oncol (Dordr).

